# Unmasking the Heart of Osimertinib: A Closer Look at Drug-Induced Cardiotoxicity

**DOI:** 10.1016/j.jaccas.2025.104401

**Published:** 2025-07-30

**Authors:** Muhammad Salman Sabri, Aheed Javaid, Hussam Al Hennawi, Hamza Muhammadzai, Vamshi Mallavarapu

**Affiliations:** a Department of Internal Medicine, Jefferson Abington Hospital, Abington, Pennsylvania, USA; b Department of Cardiology, Jefferson Abington Hospital, Abington, Pennsylvania, USA

**Keywords:** cardiomyopathy, guideline-directed medical therapy, heart failure, osimertinib

## Abstract

**Background:**

Osimertinib, a third-generation epidermal growth factor receptor tyrosine kinase inhibitor, is an effective therapy for epidermal growth factor receptor–mutated non–small cell lung cancer, but it has been associated with a higher incidence of cardiotoxicity.

**Case Summary:**

A 91-year-old woman on osimertinib 80 mg daily presented with acute dyspnea and was found to have a newly reduced left ventricular ejection fraction of 30%. Coronary angiography excluded obstructive coronary disease. Osimertinib was discontinued, and guideline-directed medical therapy (GDMT) was initiated, resulting in left ventricular ejection fraction recovery to 60%. The patient was successfully rechallenged with osimertinib while continuing GDMT, without recurrence of cardiomyopathy.

**Discussion:**

Although rare, osimertinib-induced cardiotoxicity may manifest as heart failure, arrhythmias, or QT interval prolongation.

**Take-Home Messages:**

This case underscores the potential of GDMT not only for recovery, but also for safe continuation of osimertinib. Prophylactic use of GDMT before initiating osimertinib may help reduce the risk of future cardiotoxic events.

## Graphical Abstract

**Figure:**
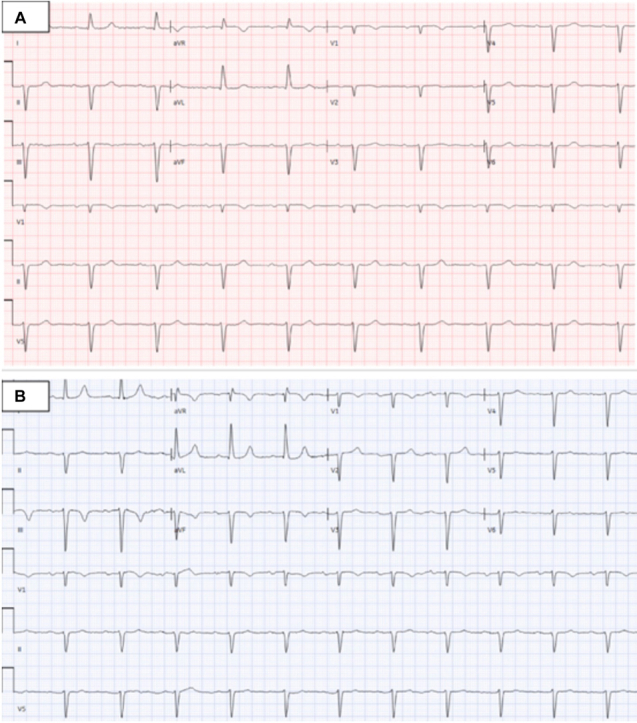

Although osimertinib is more effective than older epidermal growth factor receptor (EGFR) tyrosine kinase inhibitors (TKIs), it is associated with a higher incidence of cardiotoxicity.^1^ Risk factors for osimertinib-related cardiotoxicity include a history of smoking, hyperlipidemia, concurrent chemotherapy, and previous radiation therapy.^2^

**Take-Home Messages**

• GDMT may facilitate safe rechallenge with osimertinib in patients who develop drug-induced cardiomyopathy.
• Early initiation of β-blockers and RAAS inhibitors may support cardiac recovery and prevent recurrence of dysfunction.
• The role of GDMT as primary prophylaxis before osimertinib initiation remains an area for future investigation.

## History of Presentation

A 91-year-old woman presented with symptoms of acute-onset dyspnea, palpitations, and bilateral lower extremity swelling. Her vital signs included blood pressure of 120/80 mm Hg, heart rate of 60 beats/min, and oxygen saturation of 95% on 2-L nasal cannula. Physical examination was remarkable for decreased breath sounds bilaterally at the bases of the lungs and systolic murmur at the apex.

## Past Medical History

Her history was significant for hyperlipidemia, hypertension, and non–small cell lung cancer, treated with stereotactic radiation therapy. She was currently on osimertinib 80 mg daily, which was started 2 years prior.

## Investigations

**Figure 1:**
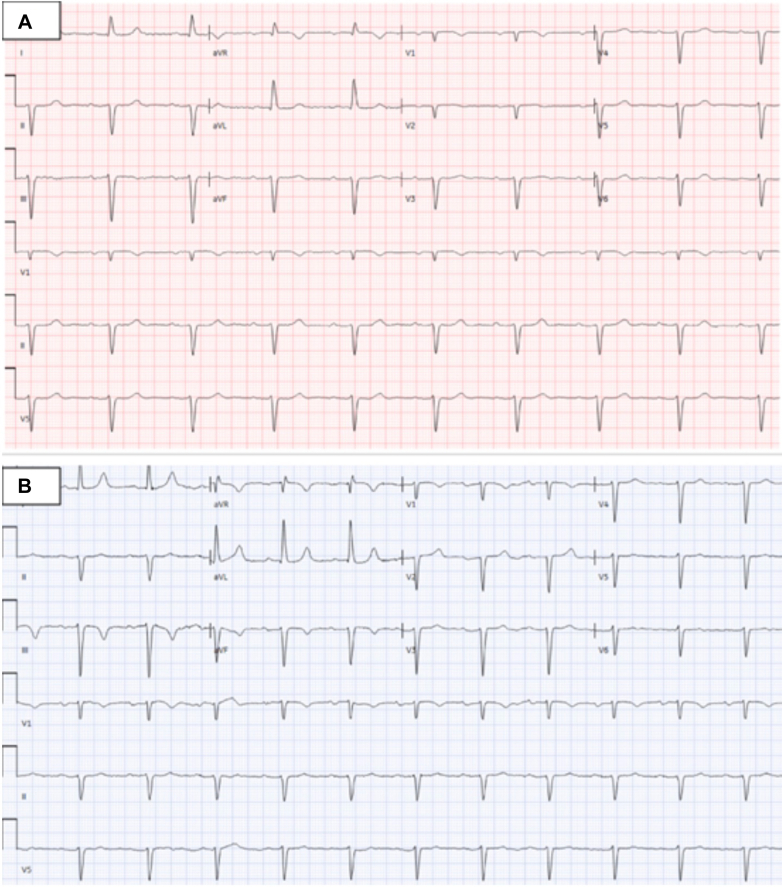
Electrocardiogram Before and After Osimertinib Baseline electrocardiogram (A) obtained before initiation of osimertinib showed normal sinus rhythm with a heart rate of 57 beats/min, first-degree atrioventricular block, left anterior fascicular block, Q waves in the anterior leads, poor R-wave progression in the anterior leads, and QT interval corrected for heart rate of 476 ms. ECG (B) obtained at the time of presentation revealed normal sinus rhythm with a heart rate of 69 beats/min, left anterior hemiblock, and a QT interval corrected for heart rate of 458 ms.

**Figure 2:**
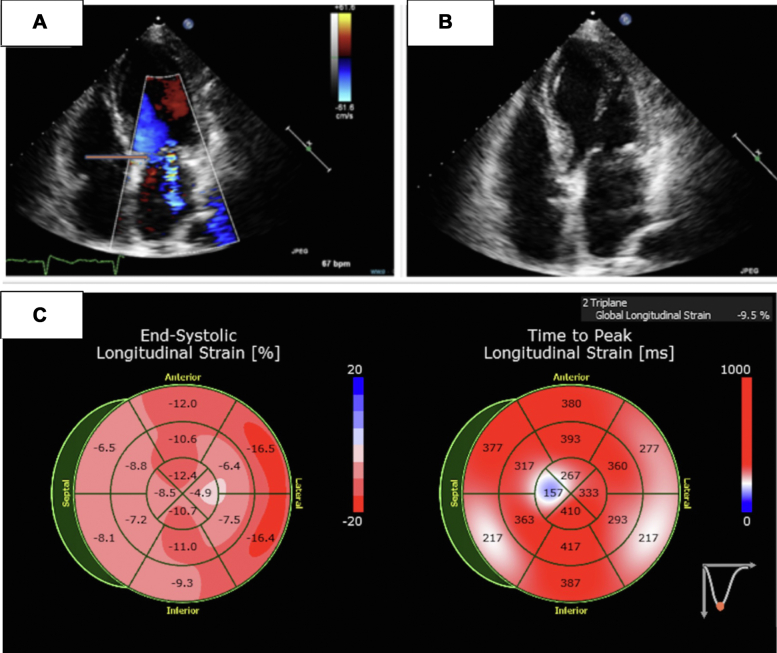
Transthoracic Echocardiogram on Osimertinib Transthoracic echocardiography reveals a dilated left ventricle (A, B) accompanied by moderate secondary mitral regurgitation, as indicated by the arrow in (A). Additionally, echocardiography (C) shows a reduced global longitudinal strain of −9.5%.

Laboratory results showed a troponin level of 49 ng/mL and N-terminal pro–B-type natriuretic peptide of 15,184 pg/mL. An electrocardiogram (ECG) obtained at the time of presentation (Figure 1B) demonstrated normal sinus rhythm with first-degree atrioventricular block, QT interval corrected for heart rate of 458 ms, left hemiblock, Q waves in anterior leads, and poor R wave progression in anterior leads, which was similar to her baseline ECG before initiation of osimertinib, QT interval corrected for heart rate 476 ms (Figure 1A). Chest x-ray revealed interstitial prominence and bilateral pleural effusions. Transthoracic echocardiography (TTE) (Figures 2A to 2C, Video 1) demonstrated newly reduced left ventricular ejection fraction (LVEF) of 30% with global hypokinesis, moderate mitral regurgitation, mild tricuspid regurgitation, and reduced global longitudinal strain −9.5 compared with a normal ejection fraction of 60% and global longitudinal strain −18 (Video 2) on initial TTE performed 6 months ago.

## Differential Diagnosis

The differential diagnosis included coronary artery disease, given the patient's history of radiation exposure 5 years ago. Additionally, considering her use of osimertinib, drug-induced cardiomyopathy was also considered as a potential cause.

## Management

**Figure 3:**
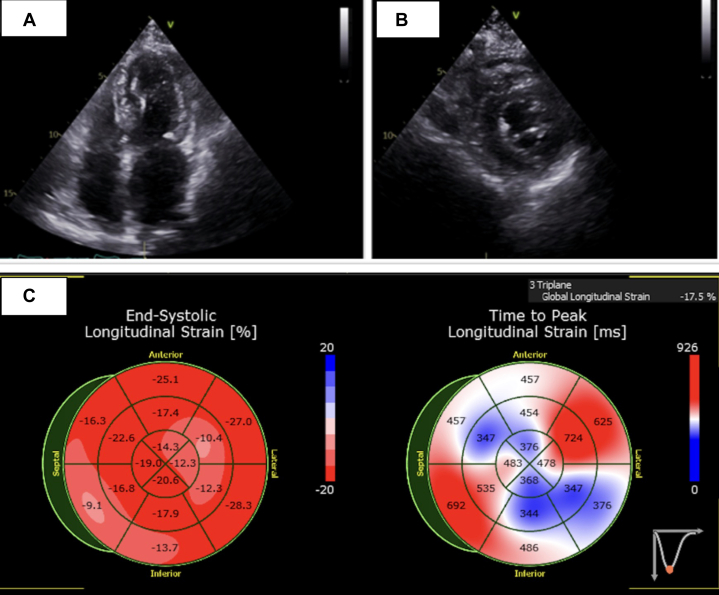
Echocardiogram After Osimertinib Discontinuation Transthoracic echocardiogram demonstrates recovery of left ventricular ejection fraction, evident in the apical 4-chamber (A) and short-axis (B) views. Global longitudinal strain also improved, measuring −17.5% (C).

The patient was treated with escalating doses of intravenous furosemide for diuresis, and a plan was made to proceed with right and left heart catheterization. Left heart catheterization demonstrated patent coronary arteries. Right heart catheterization revealed normal hemodynamics, including right atrial pressure of 1 mm Hg, pulmonary capillary wedge pressure of 6 mm Hg, and mean pulmonary artery pressure of 15 mm Hg. Given the strong suspicion of osimertinib-related cardiotoxicity, hematology was consulted and recommended discontinuation of osimertinib with ongoing surveillance to monitor for improvement in ejection fraction. After 3 months off osimertinib and initiation of a β-blocker and an angiotensin-converting enzyme inhibitor, follow-up TTE (Figures 3A to 3C) showed full recovery with normalization of ejection fraction to 60% and global longitudinal strain to −17.5 (Video 3). Recurrence of lung cancer was confirmed by thoracentesis of a new left pleural effusion, and the patient was rechallenged with osimertinib, starting at 40 mg daily and increasing to 80 mg after 3 months while continuing guideline-directed medical therapy (GDMT). Repeat TTE at 6 months after osimertinib reinitiation demonstrated preserved ejection fraction (Video 4).

## Discussion

Osimertinib is a novel third-generation EGFR TKI that is used in the treatment of EGFR-mutated non–small cell lung cancers. The standard daily dosage of this oral agent is 80 mg, with an increased dose of 160 mg recommended in the presence of leptomeningeal metastases. Compared with earlier-generation TKI inhibitors, osimertinib is associated with higher rates of cardiac adverse effects.^1^^,^^3^

Cardiotoxicity associated with osimertinib, though uncommon, remains an underrecognized and underreported adverse effect that can occur independently of dosage. Osimertinib has been linked to heart failure, QT interval prolongation, worsening of preexisting valvular disease, myocardial infarction, and arrhythmias, with a retrospective study reporting an incidence of 6.1%.^1^ The proposed mechanism behind osimertinib-induced cardiomyopathy includes inhibition of the EGFR TKI receptor, which normally provides myocardial protection against sympathomimetic injury, along with concurrent inhibition of the HER2 receptor—resulting in cardiotoxic effects similar to those seen with trastuzumab.^1^^,^^2^ β-Blockers may offer therapeutic benefit in this setting by mitigating sympathetic overstimulation that is due to the loss of EGFR-mediated cardioprotection. Moreover, osimertinib may interfere with potassium and sodium channel activity, contributing to QT interval prolongation, and has also been implicated in calcium channel inhibition, which can further predispose patients to arrhythmias.^3^

Osimertinib-induced cardiotoxicity may manifest either symptomatically, through heart failure symptoms or arrhythmias, or asymptomatically, as evidenced by changes in pretreatment and posttreatment ECG and echocardiographic findings. These changes may suggest a new onset of QT interval prolongation or a reduction of 10% or more in LVEF below the normal limit.^4^

The primary management approach for osimertinib-related cardiotoxicity involves discontinuation of the drug, coupled with the initiation of GDMT to facilitate further recovery of LVEF.^1^, ^2^, ^3^ Given the relative frequency and severity of cardiac adverse effects associated with osimertinib compared with other TKIs, it is strongly recommended to implement cardiac monitoring, including ECG and echocardiography, for patients receiving osimertinib therapy. Notably, osimertinib-related cardiac adverse effects are independently linked to poorer survival outcomes.^3^

In this case, GDMT with a β-blocker and an angiotensin-converting enzyme inhibitor led to full recovery of ejection fraction after osimertinib-induced cardiomyopathy. Notably, the patient was successfully rechallenged with osimertinib while continuing GDMT, with no recurrence of cardiac dysfunction. This suggests that GDMT may play a vital role in enabling safe reinitiation of osimertinib following cardiotoxicity.

Although secondary prophylaxis with GDMT during rechallenge appears promising, the role of GDMT as primary prophylaxis to prevent osimertinib-induced cardiomyopathy remains unclear. Further research is needed to evaluate whether preemptive initiation of GDMT in high-risk patients could reduce the incidence of cardiotoxicity and improve overall treatment tolerance. As osimertinib use becomes more widespread, proactive cardiac monitoring and consideration of cardioprotective strategies will be essential in optimizing outcomes for patients undergoing therapy.

## Conclusions

Osimertinib is linked to cardiotoxicity, making active surveillance and collaboration with the cardiology team essential both before and during treatment. GDMT enabled successful recovery and safe rechallenge with osimertinib in a patient with cardiomyopathy. This case highlights the potential role of GDMT not only as treatment, but also as secondary prophylaxis during osimertinib therapy. Further studies are warranted to explore the utility of GDMT as primary prevention in high-risk patients receiving osimertinib.

## Funding Support and Author Disclosures

The authors have reported that they have no relationships relevant to the contents of this paper to disclose.

## Appendix

Video 1 Baseline Echocardiogram Before Osimertinib Transthoracic echocardiography demonstrates moderately to severely reduced left ventricular systolic function with global hypokinesis and regional variation, with relatively preserved motion in the basal-to-mid anterolateral and inferolateral walls. The visually estimated left ventricular ejection fraction is 25% to 30%. Left ventricular chamber size and wall thickness are normal, with grade I (mild) diastolic dysfunction. The right ventricular size and function are within normal limits. The left atrium is moderately dilated. Valvular assessment reveals mild to moderate mitral and tricuspid regurgitation as well as mild aortic regurgitation. The estimated pulmonary artery systolic pressure is 28 mm Hg, assuming a right atrial pressure of 3 mm Hg.1

Video 2 Echocardiogram Showing Left Ventricular Dysfunction on Osimertinib Transthoracic echocardiography obtained before starting osimertinib demonstrates normal left ventricular systolic function, with an estimated ejection fraction of 55% to 60%. The right ventricle appears normal in size and exhibited preserved function.

Video 3 Echocardiogram After Osimertinib Discontinuation Transthoracic echocardiography performed 3 months after discontinuation of osimertinib demonstrates normal left ventricular chamber size and mild concentric left ventricular hypertrophy. The left ventricular systolic function is normal, with no segmental wall motion abnormalities, and the visually estimated ejection fraction is 60% to 65%. Doppler findings showed an E/A reversal of the mitral inflow pattern, consistent with impaired left ventricular relaxation. Mild mitral and tricuspid regurgitation are also noted.

Video 4 Echocardiogram After Osimertinib Reinitiation on Guideline-directed Medical Therapy Transthoracic echocardiography performed 6 months after reinitiating osimertinib reveals normal left ventricular systolic function with a visually estimated ejection fraction of 60% to 65%. There is evidence of grade II (moderate) diastolic dysfunction. Mild mitral regurgitation and mild to moderate tricuspid regurgitation are present, with an estimated pulmonary artery systolic pressure of 30 mm Hg. The right ventricle is normal in both size and function.
